# Exploring Sorghum Flour as a Sustainable Ingredient in Gluten-Free Cookie Production

**DOI:** 10.3390/foods14152668

**Published:** 2025-07-29

**Authors:** Simona Bukonja, Jelena Tomić, Mladenka Pestorić, Nikola Maravić, Saša Despotović, Zorica Tomičić, Biljana Kiprovski, Nebojša Đ. Pantelić

**Affiliations:** 1 Institute of Field and Vegetable Crops, National Institute of the Republic of Serbia, Maksima Gorkog 30, 21000 Novi Sad, Serbia; simona.jacimovic@ifvcns.ns.ac.rs (S.B.); biljana.kiprovski@ifvcns.ns.ac.rs (B.K.); 2 Institute of Food Technology in Novi Sad, University of Novi Sad, Bulevar cara Lazara 1, 21000 Novi Sad, Serbia; jelena.tomic@fins.uns.ac.rs (J.T.); mladenka.pestoric@fins.uns.ac.rs (M.P.); nikola.maravic@fins.uns.ac.rs (N.M.); zorica.tomicic@fins.uns.ac.rs (Z.T.); 3 Faculty of Agriculture, University of Belgrade, Nemanjina 6, 11080 Belgrade, Serbia; sdespot@agrif.bg.ac.rs

**Keywords:** gluten-free cookies, sorghum flour, bioactive compounds, RATA method, sensory evaluation, functional food

## Abstract

In this study, whole grain sorghum flour was used to partially substitute the gluten-free flour blend in cookie formulation at 20% (C20) and 40% (C40) replacement levels. The goal was to explore its potential to improve the nutritional value and sensory appeal of cookies relative to conventional and commercially available gluten-free alternatives. Nutritional analysis revealed that cookies with added sorghum flour showed increased levels of protein, ash, and polyphenolic compounds, while maintaining favorable macronutrient profiles. Notably, several bioactive compounds, such as gallic acid, caffeic acid, and apigenin, were detected exclusively in sorghum-containing samples, suggesting enhanced functional properties. Despite these compositional changes, textural measurements showed no significant differences in hardness or fracturability compared with the control. Sensory profiling using the Rate-All-That-Apply (RATA) method demonstrated that both samples (C20 and C40) achieved balanced results in terms of aroma as well as texture and were generally well accepted by the panel. The results indicate that moderate inclusion of sorghum flour (20% and 40%) can improve the sensory and nutritional profiles of gluten-free cookies without compromising product acceptability. Sorghum thus offers a promising pathway for the development of high-quality, health-oriented, gluten-free bakery products.

## 1. Introduction

Plant-based foods, including grains, vegetables, and fruits, provide essential nutrients that support optimal physiological function. Growing consumer awareness of the health-promoting potential of certain foods has led to the development of value-added products designed not only to meet basic nutritional needs but also to reduce disease risk and improve overall well-being.

Due to its resilience to drought and high temperatures, *Sorghum bicolor* L. Moench represents a more sustainable alternative to traditional cereal crops [1,2]. Although sorghum is easier and more cost-effective to cultivate than maize, it remains underutilized on a global scale [3]. Beyond its agronomic benefits, sorghum attracts growing interest as a naturally gluten-free grain suitable for individuals with celiac disease [4,5,6,7]. Given the growing consumer demand for functional foods, sorghum continues to gain recognition as a well-established ingredient with significant potential for innovative applications.

The application of sorghum in premium food formulations has recently garnered increased attention [8,9,10], offering opportunities for innovation, product diversification, and broader global recognition of this crop as a valuable food source. Nutritionally, sorghum is comparable to corn and contains a wide spectrum of phenolic compounds that confer multiple health benefits [11]. Recent studies also highlight its rich flavonoid and phytosterol content, which can improve lipid metabolism and reduce inflammation [12,13]. Consequently, the demand for this gluten-free grain continues to rise.

The current population is plagued by obesity and overweight, which are associated with numerous health complications [14]. Sorghum can be particularly beneficial in this context, as it contains proteins and polysaccharides that are resistant to digestion [15]. Given its slow digestibility and rich polyphenolic content, sorghum flour offers potential benefits for diabetic populations and consumers seeking functional foods with antioxidant properties. Furthermore, its ability to induce a slower postprandial glycemic response is particularly advantageous for individuals with diabetes. This finding is supported by a randomized cross-over trial demonstrating that sorghum consumption effectively suppresses postprandial blood glucose increases in healthy adults [16].

Incorporating sorghum into widely consumed food categories such as bakery and confectionery products offers an effective way to promote its consumption. These product groups are frequently consumed across all age demographics, making them ideal carriers for new functional ingredients. Combining sorghum with other gluten-free flours has been shown to yield acceptable gluten-free bakery products. Prior studies have demonstrated that adding sorghum to items such as breads, cakes, and snacks can provide an appealing natural coloration without compromising sensory quality [17,18].

Cookies are one of the most commonly consumed baked goods worldwide. They are often used as snacks and, in some cases, as meal replacements. Their long shelf life and relatively low cost contribute to their widespread popularity. Wheat flour is typically used in cookie production due to its favorable structural properties imparted by gluten. However, increasing consumer awareness of nutrition has encouraged the industry to explore new, nutritionally enriched ingredients suitable for the development of gluten-free cookies.

Although the use of sorghum flour in gluten-free baked products has been previously explored, most studies have relied on simplified laboratory-scale recipes, often neglecting the practical integration of sorghum into commercial gluten-free flour blends. In addition, few studies have combined comprehensive nutritional, physicochemical, and sensory evaluations of such products, especially in comparison with existing commercial alternatives. This study addresses that gap by exploring the incorporation of whole grain sorghum flour into a commercially available gluten-free cookie formulation and assessing its impact on key quality attributes [19,20]. Furthermore, the inclusion of sorghum sourced from regional genotypes contributes to the growing emphasis on sustainable and locally adaptable food ingredients.

In addition to addressing the complex technological challenges associated with production and development, gluten-free products must also ensure a sensory profile that meets consumer acceptance. This study aimed to evaluate the potential of incorporating sorghum flour into gluten-free cookies and to assess their sensory profiles in comparison with commercially available products on the Serbian market.

## 2. Materials and Methods

Low-tannin sorghum grain was produced at the Institute of Field and Vegetable Crops (Department of Vegetable and Alternative Crops, Bački Petrovac N 50°21′; E 39°56′). The whole grain sorghum flour was obtained from a genotype previously identified as nutritionally superior within a screening of 172 restorer lines developed under European agro-climatic conditions [21]. The selected genotype exhibited a high protein content, elevated levels of 3-deoxyanthocyanidins, and a strong antioxidant potential, which made it suitable for application in a gluten-free cookie formulation [22]. Milling was performed using a hammer mill LM3100 (Perten Instruments, Huddinge, Sweden) equipped with a 0.8 mm screen opening for obtaining fine particles. Other ingredients used in the formulation of the cookies, such as a gluten-free mix, cane sugar, coconut oil, and sodium bicarbonate, were purchased from local suppliers.

### 2.1. Cookie Formulations

Gluten-free cookies were produced by substitution of a commercially available gluten-free mix (Aleksandrija Premium universal mix for breads and pastries) with whole grain sorghum flour at levels of 20% (C20) and 40% (C40) flour. A control, without sorghum flour incorporation, was also prepared and further analyzed. All raw materials are shown in Table 1, and the final recipe was adopted after a series of trial bakes.

**Figure 1:**
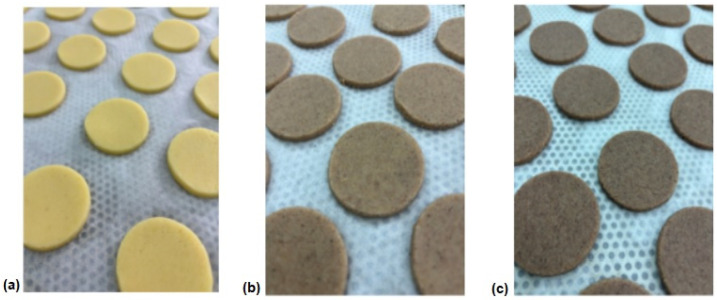
Visual appearance of shaped cookie dough prepared according to the formulations listed in Table 1. (**a**) Control; (**b**) C20; (**c**) C40.

The preparation of the dough involved mixing extra-virgin coconut oil with cane sugar and distilled water for 2 min, until a homogeneous mass was obtained. The gluten-free mixture, sorghum flour, and baking powder were mixed in certain proportions and gradually added to the prepared homogeneous mass. The resulting dough was sheeted to a thickness of 4.0 mm using a pilot-scale dough sheeter (Macpan, Thiene, Italy). The dough was shaped by pressing a mold with a diameter of 45 mm (punching method). The cookies were arranged on a tray, on which baking paper had been previously placed, and were then baked in a multi-level oven (MIWE Gusto, Arnstein, Germany) at 180 °C for 15 min. After baking, they were cooled at room temperature for 2 h. The cooled gluten-free cookies were wrapped in aluminum foil, then placed in PET containers with lids and stored in a dry and dark place for further analysis. Images of the shaped cookie doughs are presented in Figure 1.

### 2.2. Physical Properties and Color of the Cookies

Analyses of the physical indicators of the quality of the cookies included the determination of color and mass (g), the diameters of the baked cookies (d_1_ and d_2_, measured perpendicular to each other), and their thicknesses (h).

Color was determined colorimetrically on 5 randomly selected cookies (Minolta CR-400 Chroma Meter, Light Protection Tube and glass protection plate (CR-A33a)), 24 h after baking. Color values were expressed as L* (lightness/darkness), a* (redness/greenness) and b* (yellowness/blueness) [23].

The dimensions were determined using a caliper (Schubler type), on 5 randomly selected samples from each batch. The diameter of the samples was measured along two perpendicular axes (d_1_ and d_2_). Based on the measured height and diameter, the following parameters were calculated:

- eccentricity—the ratio of the diameters, serving as a measure of deviation from an ideal circular shape.
- spread factor—the ratio between the average diameter and the height of the cookies.

### 2.3. Textural Properties of Cookies

The hardness and fracturability of the cookies were determined using a TA.XT2 Texture Analyzer (Stable Micro Systems, Godalming, UK), 24 h after baking, following the “Hardness measurement of biscuits by cutting” method. Prior to analysis, the working conditions were set by selecting a 30 kg load cell and the 3-Point Bending Rig HDP/3PB accessory to measure the maximum cutting force of the sample placed on the HDP/90 metal platform.

Hardness, defined as the force at which the first fracture occurs, was measured in five replicates for each sample. The testing parameters were as follows: lever movement speed until cutting, 1 mm s^−1^; analysis speed, 3 mm s^−1^; lever return speed after cutting, 10 mm s^−1^; and distance, 5 mm.

### 2.4. Proximate Analysis

The basic chemical composition of cookies, including protein, fat, ash, and moisture content, was determined using standard AOAC methods [24]. All analyses were performed in triplicate (*n* = 3).

The proximate composition of the cookies was determined by evaluating protein (AOAC Method 920.87), fat (AOAC Method 922.06), ash (AOAC Method 923.03), and moisture (AOAC Method 925.09) contents, according to the AOAC standard methods of analysis (2000). A nitrogen-to-protein conversion factor of 6.25 was used. The total carbohydrate content was determined computationally by subtracting the sum of mass of water, protein, fat and ash in g per 100 g of a sample [25].

### 2.5. Determination of Water Activity (aw) of Biscuits

Water activity (aw) was determined by drying the samples in a temperature-controlled dryer at 105 °C until a constant weight was achieved. It was measured at 25 °C using a LabSwift-aw meter (Novasina AG, Lachen, Switzerland).

### 2.6. Polyphenolic Analysis

Qualitative and quantitative testing of polyphenolic compounds in the samples was performed by high-resolution liquid chromatography (HPLC) in three replicates (*n* = 3), on a Shimadzu Prominence device (Shimadzu, Kyoto, Japan), equipped with an LC-20AT binary pump, CTO-20A thermostat, SIL-20A automatic dispenser, and SPD-M20A detector at the University of Novi Sad, Institute of Food Technology (FINS). Separation of polyphenolic compounds was performed on a Luna C18 RP column 250 × 4.6 mm, 5 μm (Phenomenex, Torrance, CA, USA), with a C18 guard precolumn, 4 × 30 mm (Phenomenex, Torrance, CA, USA). A solvent system was used as the mobile phase: A (acetonitrile) and B (1% formic acid) at a flow rate of 1 mL min^−1^, using a linear gradient: 0–10 min from 10 to 25% A, 10–20 min linear increase to 60% A, from 20 to 30 min linear increase to 70% A. Column equilibrated to initial conditions, 10% A, 10 min with an additional 5 min for stabilization. Chromatograms were recorded in the wavelength range from 190 nm to 800 nm. For each polyphenolic compound, identification and quantification were performed at the wavelength of its absorption maximum. Based on the obtained chromatograms and calibration diagrams of standard solutions of polyphenolic compounds, the concentrations of the identified compounds were expressed as mg 100 g^−1^ of a sample.

### 2.7. Amino Acid Analysis

Amino acid analysis was determined by ion exchange chromatography using an automatic amino acid analyzer, Biochrom 30+ (Biochrom, Cambridge, UK) in two replicates (*n* = 2) at the University of Novi Sad, Institute of Food Technology (FINS), according to the method described by Spackman et al. [26]. The technique is based on the separation of amino acids with the help of strong cation exchange chromatography, followed by the ninhydrin reaction and photometric detection at 570 nm (except for proline, which is detected at 440 nm). The samples were initially hydrolyzed with 6M of HCl (Merck, Darmstadt, Germany) at 110 °C for 24 h. Similarly, alkaline hydrolysis with 4 M of NaOH (Merck, Germany) was used to determine tryptophan. After hydrolysis, the samples were cooled to room temperature and dissolved in 25 mL of sodium loading buffer, pH 2.2 (Biochrom, Cambridge, UK). Samples were filtered through a 0.22 µm pore size PTFE syringe filter (Plano GmbH, Wetzlar, Germany), transferred to vials (Agilent Technologies, Santa Clara, CA, USA), and stored in a refrigerator prior to analysis. Amino acids were identified by comparing the retention time of identified amino acids with the retention times of amino acid standards (amino acid standard solution, Sigma-Aldrich, St. Louis, MO, USA). The results were expressed as g 100 g^−1^ of a sample [27].

### 2.8. Sensory Evaluation

Cookie samples of various formulations, including the gluten-free control and the experimental versions containing whole grain sorghum flour (C20 and C40), were assessed as a part of the sensory analysis. In addition, two commercially available products were included, providing a broader representation of similar products on the domestic market (Table 2). The inclusion of commercial samples enabled a more comprehensive understanding of the sensory properties. By investigating the influence of different formulations on the sensory profile of the finished product, valuable insights were gained that could serve as a basis for future development and optimization of the existing recipe.

The sensory evaluation of the cookies was performed at the University of Novi Sad, Institute of Food Technology (FINS). The panel consisted of 15 assessors, aged between 30 and 60 years, with prior experience in the sensory evaluation of cookies. All assessors received additional training in the RATA (Rate-All-That-Apply) method and in the specific sensory attributes relevant to this product category. All participants provided informed consent prior to participation, and the sensory evaluation was approved by the Ethics Committee of the Institute of Food Technology, University of Novi Sad, under decision no. 24-53-3.

The RATA (Rate-All-That-Apply) method enables efficient, modern, and rapid sensory profiling of products. It allows assessors to simultaneously evaluate a wide range of predefined sensory attributes, providing a comprehensive understanding of the product’s sensory characteristics. This approach is particularly valuable in product development and recipe optimization, as it generates feedback that supports improvements in overall product quality. By only selecting the relevant attributes for each sample, panelists streamline the evaluation process and minimize the risk of error. Furthermore, the RATA method can stimulate discussion among assessors, leading to deeper insights and a more nuanced understanding of the sensory profile.

A list of 47 sensory attributes was compiled to characterize the individual cookie samples (Supplementary Table S1). Assessors were instructed to select all relevant attributes from the list and rate their intensity on a 5-point scale, where 0 indicated absence of the attribute, 1 indicated a very weak presence, and 5 indicated an extremely strong presence. Testing was carried out in two sub-sessions of 45 min each, with two replicates conducted in randomized order. Samples were coded with random three-digit numbers and served at room temperature (22 ± 2 °C) in transparent plastic containers with lids to ensure optimal sensory perception. After evaluating each sample, assessors took a 60 s break, during which they refreshed their palate with mineral water. The sensory evaluation was carried out in individual cabinets for the sensory assessment, in the Laboratory for Sensory Assessment, the Laboratory for Sensory Evaluation in accordance with the SRPS EN ISO 8589 standard [28].

### 2.9. Statistical Analysis

The XLSTAT software version 2023.3.1 (Addinsoft, New York, NY, USA) was used to statistically process the mean values for the analyzed parameters. The significance of the differences between the sample mean values was assessed using an analysis of variance (ANOVA) and a Tukey’s honest significant difference test (*p* < 0.05). Based on the Principal Component Analysis (PCA), the sensory profiles of the samples were evaluated, allowing for the identification of patterns and relationships among the different sensory attributes and product formulations.

## 3. Results and Discussion

This study comprehensively evaluated the potential of whole sorghum flour as a functional ingredient in gluten-free cookie formulations through physicochemical, nutritional, and sensory analyses. The incorporation of sorghum flour aimed to enhance not only the nutritional profile but also the sensory quality of gluten-free baked goods, a category of products often challenged by texture and flavor limitations.

### 3.1. Physical and Color Characteristics

The evaluation of color and physical properties in cookies containing varying proportions of whole sorghum flour provides insights into how different substitution levels affect overall product quality (Table 3).

Color measurements revealed significant differences between samples. The control sample exhibited the highest L* values (lightness), while the 40% sorghum sample showed the lowest, indicating a darker appearance. All samples containing sorghum flour demonstrated significantly higher a* values, while the values of b* (yellow–blue coordinate) decreased with increasing sorghum flour content, with the control sample showing the highest b* value (34.59), followed by C20 (17.52), and C40 (14.92), reflecting that the addition of sorghum flour results in a decrease in the b* values. These changes in color parameters likely result from the natural pigments present in whole sorghum flour, including anthocyanins and phenolic compounds, which intensify redness and yellowness in baked products [22,29]. As shown in Table 4, the total color difference (∆E) between the control and the sorghum-based samples is substantial. An ∆E value of 28.54 between the control and C20, and 34.29 between the control and C40, indicates highly perceptible visual differences. These values far exceed the commonly cited threshold of 2–3 for perceptibility, and even the ∆E of 5.84 between C20 and C40 is considered noticeable by most observers. These findings confirm that the inclusion of sorghum flour significantly alters the visual appearance of the cookies, mainly due to its pigment content. This effect is consistent with observations in similar baked products, where ∆E values above 10 are typically associated with a strong and recognizable difference in product color [30].

All cookie formulations exhibited comparable physical dimensions, indicating similar dough behavior during baking. The results for eccentricity, defined as the ratio d1/d2 and used to measure deviation from an ideal circular shape, confirmed a uniform circular form across all samples. The spread ratio, calculated as the diameter-to-thickness ratio, was also consistent among the formulations. Additionally, the incorporation of sorghum flour significantly affected the weight of the cookies compared to the control. A high spread ratio, typically influenced by dough flow and expansion during baking, is considered a desirable quality characteristic in cookies [31]. In this study, the addition of whole sorghum flour did not significantly affect the spread ratio, except in sample C20, where a slight decrease was observed. Badi and Hoseney [32] reported that cookies with 100% sorghum did not spread during baking, while Rai et al. [33] found that incorporating sorghum flour into a gluten-free formulation with rice flour tended to limit cookie spread. In general, the inclusion of wholegrain flour or bran in cookie formulations has been shown to cause either a slight decrease or no significant effect on the spread factor [34,35]. This effect is likely related to their content of fiber, which can absorb large amounts of water, thereby reducing the water available to dissolve sugar in the dough. As a result, the initial dough viscosity increases, leading to reduced cookie spread during baking.

### 3.2. Textural Properties

In addition to physical characteristics, textural properties such as hardness and fracturability are key quality indicators in cookie evaluation. Cookie hardness must be sufficient to withstand transport and storage, while remaining acceptable for consumption in terms of bite and mouthfeel. According to Mitevski et al. [36], the ideal hardness for cookies with balanced fat and sugar contents ranges between 2000 and 3000 g. Variations in this property are largely influenced by the composition of the samples, particularly their proportion of lipids, proteins, and starch. As is presented in Table 5, the incorporation of whole sorghum flour did not result in statistically significant changes in either hardness or fracturability when compared to the control (*p* < 0.05). These findings suggest that partial replacement with sorghum flour does not compromise structural integrity, thereby maintaining the mechanical stability of the final product.

### 3.3. Proximate Composition

The results of the proximate composition of cookies are shown in Table 6. All tested samples had a fat content of approximately 20%. However, the addition of 40% sorghum flour had a statistically significant effect (*p* < 0.05) on the change in fat content, from 20.02% in the control to 20.96% in the C40 sample. The observed variation in protein content with increasing sorghum flour suggests that its inclusion enhances the nutritional value of the cookies compared to the control. Specifically, protein content increased from 3.52% in the control to 4.41% in the C20 sample and 4.24% in the C40 sample. Although the C20 sample showed the highest protein level, both sorghum-containing variants exhibited statistically significant differences compared to the control (*p* < 0.05), suggesting that sorghum flour contributed valuable plant-based proteins. A significant difference in carbohydrate content was also noted in sorghum-containing samples relative to the control. The most pronounced changes in the examined samples are observed in the ash content, which reflects the total mineral content. Ash values increased progressively with sorghum addition from 0.44% in the control, to 0.57% in the C20 sample and reaching 0.70% in the C40 sample. These differences were statistically significant (*p* < 0.05), indicating that sorghum flour was a richer source of minerals compared to the gluten-free mix. This result aligns with the literature findings that sorghum contains higher levels of minerals such as iron, phosphorus, and magnesium, and its incorporation into bakery products may enhance their functional and nutritional properties [22]. The marked rise in ash content confirms sorghum’s potential in contributing to dietary mineral intakes through commonly consumed products like cookies. A slight decrease, not even statistically different, in moisture content was observed with higher levels of sorghum flour (from 4.21% in the control to 4.02% in the 40% sample), possibly due to differences in water-binding capacity. Moisture content and water activity (*aw*) provide important insights into the shelf life of sorghum-containing biscuits, as *aw* is considered a critical control parameter and reflects the product’s stability, potential for microbial growth, and overall shelf life [36]. In this study, the measured *aw* values ranged from 0.46 in the control sample to 0.49 in sample C20 and 0.48 in sample C40, indicating good storability and the microbiological safety of all final products. This modest rise in *aw* values observed in sorghum-containing cookies could be attributed to the presence of hydrophilic dietary fiber and polyphenols in whole grain sorghum flour, which can interact with water and affect its availability within the food matrix [35]. These interactions may influence both shelf-life and texture, warranting further investigation. This shift in macronutrient composition, particularly the increased ash and protein contents, highlights sorghum’s potential to improve the micronutrient density and functional profile of gluten-free baked products.

### 3.4. Polyphenolic Profile

A clear increase in specific polyphenolic compounds is presented in Table 7, correlating directly with the level of sorghum flour substitution. In comparison to the control product, samples containing the integral sorghum flour exhibited the presence of four polyphenolic compounds absent in the control sample: *p*-coumaric acid, cinamic acid, naringin, and apigenin. These results highlight sorghum’s potential as a functional ingredient in gluten-free product formulations, contributing to improved health-related properties. Changes in the contents of chlorogenic, sinapic, and ferulic acids do not show statistically significant differences in the examined samples. However, statistically significant differences are observed in the contents of *p*-hydroxybenzoic and gallic acids with the addition of whole sorghum flour. In addition, the contents of luteolin, apigenin, *p*-coumaric, and cinnamic acids show statistically significant differences compared with the control, which indicates an increase in the biological value of the cookies with the addition of integral sorghum flour. In alignment with our study’s findings, Yousif et al. [37] and Renzella et al. [38] similarly revealed that using sorghum flour markedly enhanced the total polyphenolic content and antioxidant capacity of the final product. Through statistical analysis, it was confirmed that the addition of integral sorghum flour significantly affected the phenolic profile of the analyzed samples, whereby the C40 sample generally shows statistically significant changes compared with the control sample (*p* < 0.05). The presence of bioactive compounds such as gallic acid, caffeic acid, and apigenin, particularly in the 40% sorghum sample, suggests an improved antioxidant potential and possible functional food applications. This is consistent with prior work demonstrating that these phenolic compounds derived from *Sorghum bicolor* exhibit significant radical-scavenging and anti-inflammatory activity [39].

### 3.5. Amino Acid Profile

Cereals generally lack certain essential amino acids, making it important to assess amino acid composition in the final product from both nutritional and sensory perspectives. Moreover, gluten-free products are often considered nutritionally inferior, especially in terms of protein quality and balance of essential amino acids. Since our control sample is based on a carbohydrate-rich ingredient, the introduction of sorghum flour is expected to improve the protein content. However, this increase is not fully reflected in the amino acid composition. Indeed, the total amino acid content increased from 3.30 g/100 g in the control to 4.15 g/100 g in the C20 sample and 4.25 g/100 g in the C40 sample (Table 8). Statistical analysis confirmed that both C20 and C40 samples showed significantly higher total amino acid content compared with the control (*p* < 0.05), while the difference between C20 and C40 was not significant. Significant increases in the concentrations of several specific amino acids, especially glutamic acid, leucine, proline, tyrosine, and arginine, were linked to higher amounts of sorghum grain. However, the increase in total protein content was not proportionally reflected in all amino acids. For example, lysine, an essential amino acid typically limited in cereals, showed only a slight increase in the C40 sample from 0.12 g/100 g in the control to 0.15 g/100 g. These results suggest that sorghum flour may still have only a limited potential to address important amino acid imbalances in cereal-based products, even while it improves the overall amino acid profile.

### 3.6. Sensory Profile of Cookies

Sensory analysis of cookies formulated with varying proportions of whole sorghum flour (20% and 40%) was conducted using the RATA methodology, providing detailed insights into the sensory profiles of the products. The collected data were processed using multiple analytical techniques and presented through various visualization tools to facilitate interpretation of how the inclusion of whole sorghum flour influences the sensory profile. The results of the evaluation using the RATA method are presented through three complementary forms of visualization: a word cloud, which highlights the frequency of individual sensory descriptors; a spider (radar) plot, which enables comparison of attribute intensities among the samples; and a two-dimensional positioning diagram, which displays the arrangement of samples in relation to dominant sensory characteristics, accompanied by a descriptive profile of each samples.

**Figure 2:**
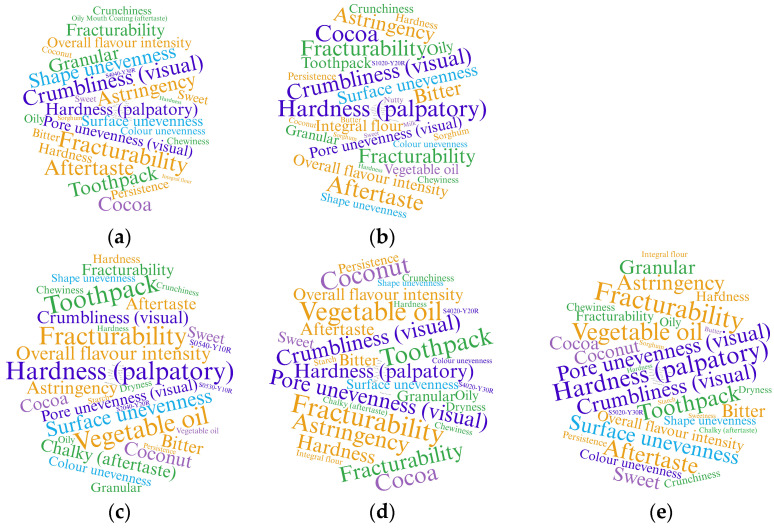
Word clouds illustrating the profile of the samples based on frequency of RATA attributes: (**a**) Commercial sample 1; (**b**) Commercial sample 2; (**c**) Control; (**d**) C20; (**e**) C40.

“Word clouds” presented in Figure 2 visually display the data, allowing quick identification of the key attributes within the analyzed samples. By examining these attributes, the dominant characteristics of each sample are apparent, facilitating the differentiation of similarities and differences among them. Word clouds offer an intuitive method for representing complex data and communication between researchers and production teams, with the size of each word reflecting its frequency or relative importance.

Textural attributes, such as hardness, surface unevenness, tactile firmness, and crumbliness, appeared most frequently, as indicated by their prominence in the word clouds. This suggests their central role in shaping the sensory profile of all evaluated samples. Aroma-related descriptors, particularly coconut notes attributed to the presence of coconut oil, were commonly selected for the experimental samples, indicating a sensory distinction from commercial products. Additionally, attributes such as sweet aroma, dryness, aroma persistence, and chalky aftertaste show moderate frequency, implying a significant but not dominant role in the description of the samples. Commercial samples also have a moderate frequency of attributes that characterize the bitter taste and astringency of these samples. In contrast, color shade descriptors (S2040-Y20R; S1020-Y20R; S5020-Y30R; S4040-Y30R) were less frequently selected, suggesting lower relevance or weaker perceptibility in differentiating the samples. The prevalence of texture-related terms further emphasizes the importance of mechanical attributes in gluten-free baked product acceptability, often outweighing minor flavor differences.

**Figure 3:**
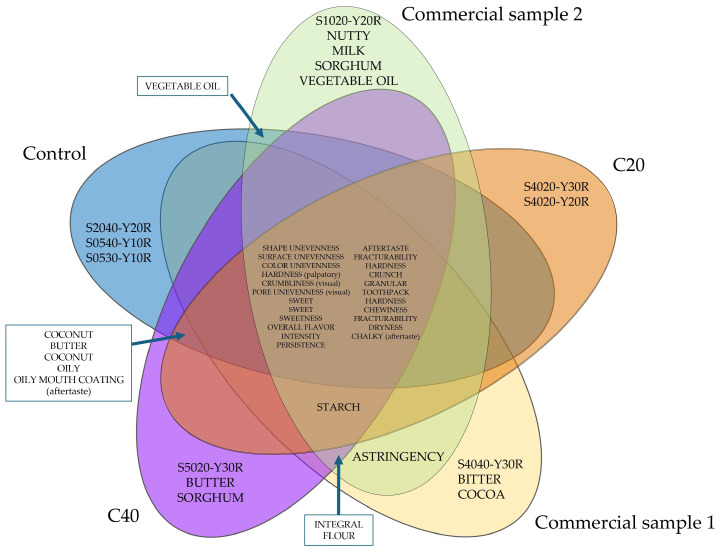
Venn diagram illustrating sensory attributes selected by at least 30% of the panelists to describe the cookies.

As illustrated in the Venn diagram (Figure 3), many high- and medium-frequency sensory attributes were shared across the samples, while several distinct features allowed clear differentiation. This analysis, combined with the first visualization of the results, provided valuable information about the overlapping sensory characteristics. The Venn diagram presents sensory attributes that are unique to, or shared among, the evaluated samples. Overlapping areas indicate common attributes, while non-overlapping areas depict attributes unique to individual samples. This mode of presentation facilitates a clearer understanding of the similarities and differences between the sensory profiles of the samples. Despite variation in attribute intensity, the overlap of core descriptors suggests that all samples meet a baseline standard of sensory quality. Accordingly, all samples are characterized in terms of appearance by unevenness of shape and surface, sweet smell, as well as persistence and sweet aroma. Samples enriched with sorghum flour (C20 and C40) were primarily differentiated by their distinct color tones and the presence of starch-like and sorghum-specific aromas, particularly in C40. Commercial sample 1 was distinguished by its unique color shade and the pronounced presence of bitter taste and aroma. In contrast to it, commercial sample 2 was separated based on nutty, milky, and grainy aromas as well as the present aroma of vegetable oil, in addition to its color shade. Astringency emerged as a shared negative attribute in both commercial gluten-free samples, further highlighting their sensory limitations. Consequently, the Venn diagram analysis confirmed that while some sensory descriptors were universal across formulations, subtle differences in aroma and texture, particularly those introduced by sorghum, enabled clear product differentiation and positioning.

**Figure 4:**
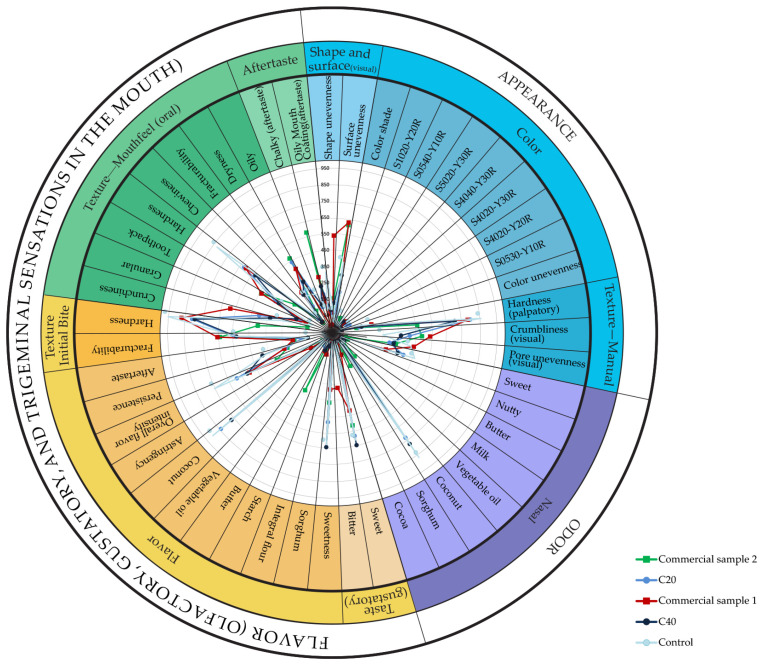
Spider plot with intensity and frequencies.

In order to show the intensity and frequency of individual attributes as clearly as possible, a spider graph was created, as shown in Figure 4. A weighted measure, reflecting the frequency of perception and perceived intensity of each attribute, was calculated by multiplying the sum of intensity values by their corresponding frequencies [40]. This visualization provides a comprehensive overview of sensory profiles, thereby facilitating direct comparisons across all evaluated samples.

In addition to attributes that positively contributed to the sensory profile and overall likeability of the samples, undesirable characteristics typical for this product category, such as bitterness and astringency, were also evaluated. Commercial sample 1 was primarily characterized by bitterness, whereas astringency was a recurring negative attribute in both commercial gluten-free products. Moreover, the commercial gluten-free cookies exhibited notable visual and textural deficiencies, which further diminished their overall sensory appeal. Although color shades differentiate all samples from one another, a comprehensive overview is provided by the diagram, where color intensities are quantitatively represented. Differences in textural and aroma-related attributes were observed across the samples. The inclusion of 20% sorghum flour enhanced both texture and aroma compared with the control, contributing to a more balanced sensory profile. However, the 20% sorghum flour samples showed a slight reduction in sweet aroma intensity. Coconut-related aroma notes, positively perceived by evaluators, were absent in the commercial gluten-free samples, further distinguishing them from the experimental formulations. Overall, the spider plot thus supports the conclusion that sorghum flour not only preserves but enhances sensory complexity, regarding favorable aroma and textural attributes.

**Figure 5:**
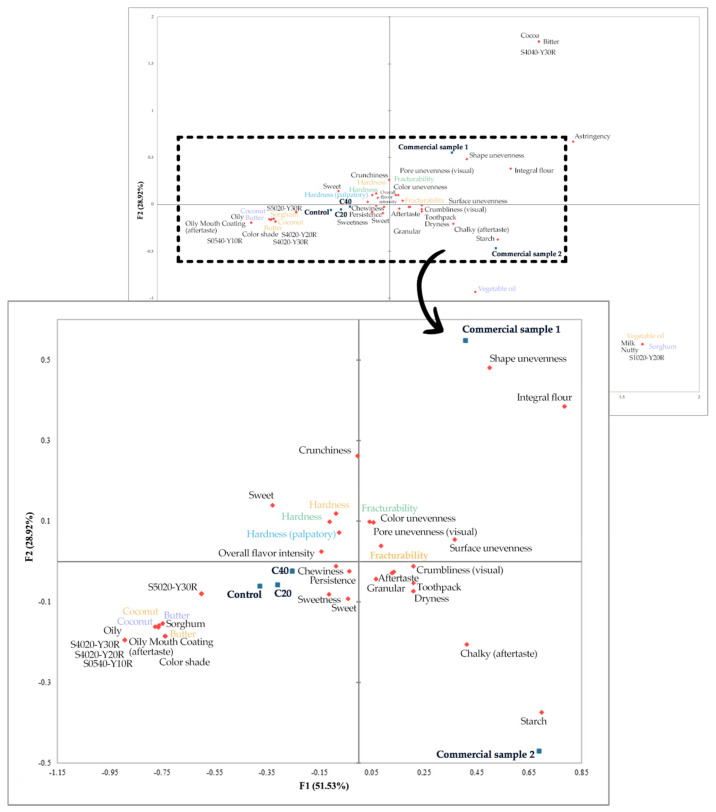
Biplot of cookie samples with attribute differentiation using RATA method.

Through the analysis of the RATA data, the key characteristics of the gluten-free cookies were identified, enabling the differentiation of samples via visualization in a biplot of the principal components PC1 and PC2 (Figure 5). The PCA results revealed that the first two principal components (PC1 and PC2) together accounted for 80.45% of the total variance, providing a robust summary of the sensory data. The PCA (*p* < 0.05) revealed that PC1 explained 51.53% of the variance, while PC2 contributed 28.92%, successfully capturing most of the total variance in the analyzed samples and variables.

Attributes such as shape and surface unevenness were strongly associated with positive PC1 values, indicating a direct link between visual imperfections and this component. Products exhibiting greater shape irregularities and surface unevenness were located in the positive part of PC1, reflecting these visual imperfections. Sweet aroma and crispness were associated with negative values of both PC1 and PC2. Positive sensory characteristics related to sweet aroma and crispness were observed in samples positioned closer to the center of the biplot, whereas samples in the positive region of PC1 showed a weaker expression of these traits. Bitter taste and astringency were associated with positive values of the PC2 component. Samples scoring higher on this axis tended to exhibit more pronounced bitterness, potentially impacting overall consumer acceptance.

Commercial sample 1 (0.407, 0.549) is located in the positive part of both PC1 and PC2, indicating high results in terms of crispness and bitterness. It also exhibits a pronounced non-uniformity in shape and surface. Commercial sample 2 (0.688, −0.470) is located in the positive quadrant of PC1, reflecting significant unevenness in shape and surface. Its negative value on PC2 suggests a weaker sweet aroma and crispness. This sample exhibits significant visual imperfections and lower scores in textural attributes, which may affect the overall consumption experience. Additionally, this sample shows notable visual imperfections and lower scores for textural attributes, which may negatively impact the overall consumption experience. The gluten-free cookie with 20% sorghum flour (C20) (−0.311, −0.058) is situated near the center of the biplot, indicating balanced characteristics in terms of shape and surface non-uniformity. This sample exhibited an improved sweet aroma and crispness, reflected by its low PC2 value, suggesting that the addition of 20% whole sorghum flour enhanced texture and aroma, resulting in a well-balanced product. The gluten-free cookie with 40% sorghum flour (C40) (−0.253, −0.025) is also near the biplot center, showing slightly higher sweet aroma and crispness values. Although it has a good sensory profile, a slight reduction in sweet aroma may affect its overall acceptability. The control sample, positioned in the negative quadrant of both PC1 and PC2, demonstrated a well-balanced profile, combining favorable visual, textural, and aromatic attributes. This sample shows optimal results in terms of sweet aroma and crispiness, so it can be seen as a reference sample for quality with an optimal ratio of visual quality, texture, and taste, setting a high standard for product excellence. These findings confirm that moderate inclusion of sorghum flour (20–40%) can produce gluten-free cookies with sensory characteristics that are competitive with, or even superior to, existing products on the market. Comparable sensory improvements in cookie formulations have also been observed with the incorporation of whole grain ancient wheat sourdough, as demonstrated by Maravić et al. [41], highlighting the relevance of ingredient selection and processing techniques in shaping consumer acceptance.

Based on the analysis of the data obtained by the sensory assessment, with the application of the RATA method, the addition of 20% or 40% of integral sorghum flour significantly improved the textural and aromatic properties of the product. Samples C20 and C40 exhibited balanced characteristics in terms of sweet aroma and crispness. In contrast, the commercial samples were characterized by a high non-uniformity of shape and surface, as well as a pronounced bitter taste, indicating a need for further optimizations to achieve better sensory properties. The control sample served as a reference point with an optimal balance of visual quality, texture and aroma, while leaving sufficient sensory space that highlights the potential of sorghum as a valuable ingredient in the development of quality gluten-free cookie samples.

## 4. Conclusions

In light of accelerating climate change and increasing consumer demand for nutritious and sustainable foods, the integration of underutilized crops such as sorghum into food systems presents both environmental and nutritional advantages. Sorghum’s natural resilience, gluten-free nature, and rich profile of polyphenolic compounds make it a promising ingredient for developing functional bakery products tailored to health-conscious and gluten-intolerant populations.

This study demonstrated that substituting a conventional gluten-free flour blend with 20 and 40% whole grain sorghum flour significantly improved the nutritional composition of cookies, particularly in terms of protein, ash, and polyphenol content in tested products. Importantly, these enhancements were achieved without compromising the key sensory attributes of cookies. Both C20 and C40 samples displayed favorable texture and aroma profiles and were well-received in sensory testing. In contrast, commercial gluten-free products exhibited several sensory drawbacks, highlighting the potential of sorghum-based formulations to raise quality standards in this product category.

These results confirm that moderate inclusion of sorghum flour can yield gluten-free cookies with nutritional and sensory characteristics that are not only comparable to traditional formulations but, in some aspects, even superior. The findings underscore sorghum’s promise as a sustainable raw material for the development of next-generation gluten-free bakery products. Future research should focus on further optimizing sorghum-based formulations, scaling up production, and exploring consumer acceptance in broader markets to support its widespread use in commercial gluten-free applications.

## Figures and Tables

**Table 1:** Composition of raw materials used in gluten-free cookies in grams (g).

Ingredients	Control	C20	C40
gluten-free mix	150	120	90
sorghum flour	/	30	60
cane sugar	50	50	50
coconut oil	52.50	52.50	52.50
sodium bicarbonate	0.75	0.75	0.75
water	55	55	55

**Table 2:** Commercial samples of gluten-free cookies included in the sensory analysis.

Sample	Description	Ingredients
Commercial sample 1	Cocoa biscuit PUSA (lactose-free, gluten-free, casein-free)	Millet flour, rice flour, corn flour, corn starch, vegetable fat, cocoa powder with reduced fat content (10%), sugar, emulsifier (soy lecithin), salt, raising agent (ammonium bicarbonate), thickener (guar gum).
Commercial sample 2	Buckwheat cookies (lean, lactose-free, casein-free, preservative-free)	Rice flour, corn starch, sugar, millet flour, palm oil, buckwheat flour (6.5%), corn flour, guar bean flour, raw soy lecithin, leavening agent (ammonium bicarbonate), salt.

**Table 3:** Physical properties and color of the analyzed cookies ($\bar{X}$ ± SD). Statistically significant differences (*p* < 0.05) are shown with different superscript-letters (a, b, c).

Sample	L* (D65)	a* (D65)	b* (D65)	Height (mm)	Weight (g)	Eccentricity	Spread Factor
Control	73.18 ± 0.62 ^a^	0.31 ± 0.41 ^a^	34.59 ± 0.76 ^a^	5.54 ± 0.19 ^a^	7.55 ± 0.05 ^a^	1.01 ± 0.01 ^a^	7.74 ± 0.28 ^a^
C20	51.78 ± 1.23 ^b^	8.38 ± 0.33 ^b^	17.52 ± 0.43 ^b^	5.57 ± 0.08 ^b^	7.49 ± 0.06 ^b^	1.00 ± 0.02 ^a^	7.56 ± 0.06 ^b^
C40	46.78 ± 0.96 ^c^	9.91 ± 0.37 ^c^	14.92 ± 0.26 ^c^	5.55 ± 0.17 ^a^	7.52 ± 0.17 ^c^	1.00 ± 0.01 ^a^	7.83 ± 0.24 ^a^

**Table 4:** Total color difference (∆E) between cookie samples based on CIE-Lab values.

Comparison	∆E Value
Control *vs.* C20	28.54
Control *vs.* C40	34.29
C20 *vs.* C40	5.84

**Table 5:** Hardness and fracturability ($\bar{X}$ ± SD) of tested gluten-free samples. No significant differences (*p* > 0.05); values are marked with the same superscript letter (a).

Sample	Hardness (g)	Fracturability (mm)
Control	2145.76 ± 81.50 ^a^	36.52 ± 0.20 ^a^
C20	2373.60 ± 157.95 ^a^	36.67 ± 0.20 ^a^
C40	2340.32 ± 190.63 ^a^	36.56 ± 0.31 ^a^

**Table 6:** Chemical composition ($\bar{X}$ ± SD) and energy value of tested samples. Statistically significant differences (*p* < 0.05) are shown with different superscript-letters (a, b, c).

Sample	Moisture (%)	Ash (%)	Fat (%)	Protein (%)	Carbohydrates (%)	Energy Value (kcal 100 g^−1^)
Control	4.21 ± 0.04 ^a^	0.44 ± 0.01 ^a^	20.02 ± 0.09 ^a^	3.52 ± 0.02 ^a^	71.81 ± 0.11 ^a^	481.47
C20	4.12 ± 0.07 ^a^	0.57 ± 0.01 ^b^	20.23 ± 0.13 ^a^	4.41 ± 0.02 ^b^	70.68 ± 0.20 ^b^	482.37
C40	4.02 ± 0.02 ^a^	0.70 ± 0.00 ^c^	20.96 ± 0.08 ^b^	4.24 ± 0.02 ^c^	70.08 ± 0.09 ^b^	485.94

**Table 7:** Content of polyphenolic compounds ($\bar{X}$ ± SD) in tested samples (mg 100 g^−1^ sample). Statistically significant differences (*p* < 0.05) are shown with different superscript-letters (a, b, c).

Compounds	Control	C20	C40
*p*-Hydroxybenzoic acid	0.27 ± 0.00 ^a^	0.35 ± 0.00 ^b^	0.43 ± 0.00 ^c^
Gallic acid	1.01 ± 0.01 ^a^	1.33 ± 0.03 ^b^	1.53 ± 0.00 ^c^
Protocatechuic acid	0.20 ± 0.00 ^a^	0.23 ± 0.00 ^b^	0.33 ± 0.00 ^c^
Chlorogenic acid	0.23 ± 0.00 ^a^	0.23 ± 0.00 ^a^	0.23 ± 0.00 ^a^
Caffeic acid	0.07 ± 0.00 ^a^	0.59 ± 0.00 ^b^	1.33 ± 0.03 ^c^
Vanillic acid	0.05 ± 0.00 ^a^	0.28 ± 0.00 ^b^	0.78 ± 0.00 ^c^
Syringic acid	0.33 ± 0.00 ^a^	0.32 ± 0.00 ^a^	0.36 ± 0.00 ^a^
Sinapic acid	0.15 ± 0.00 ^a^	0.16 ± 0.00 ^a^	0.15 ± 0.00 ^a^
*p*-Coumaric acid	*n.d.* ^a^	0.04 ± 0.00 ^b^	0.28 ± 0.00 ^c^
Cinamic acid	*n.d.* ^a^	0.05 ± 0.00 ^b^	0.21 ± 0.00 ^c^
Ferulic acid	0.43 ± 0.00 ^a^	0.47 ± 0.03 ^a^	0.49 ± 0.00 ^a^
Luteolin	0.05 ± 0.01 ^a^	0.16 ± 0.00 ^b^	0.31 ± 0.00 ^c^
Naringin	*n.d.* ^a^	0.04 ± 0.00 ^b^	0.09 ± 0.00 ^c^
Apigenin	*n.d.* ^a^	0.05 ± 0.00 ^b^	0.09 ± 0.01 ^c^

*n.d.—not detected.*

**Table 8:** Amino acid content ($\bar{X}$ ± SD) in tested samples (g 100 g^−1^ sample). Statistically significant differences (*p* < 0.05) are shown with different superscript-letters (a, b, c).

Compounds	Control	C20	C40
Aspartic acid	0.34 ± 0.01 ^a^	0.40 ± 0.01 ^b^	0.38 ± 0.00 ^b^
Threonine	0.13 ± 0.00 ^a^	0.15 ± 0.00 ^b^	0.15 ± 0.00 ^b^
Serine	0.20 ± 0.01 ^a^	0.24 ± 0.01 ^b^	0.26 ± 0.01 ^b^
Glutamic acid	0.69 ± 0.02 ^a^	0.85 ± 0.02 ^b^	0.86 ± 0.00 ^b^
Proline	0.17 ± 0.01 ^a^	0.22 ± 0.00 ^b^	0.25 ± 0.00 ^c^
Glycine	0.17 ± 0.00 ^a^	0.18 ± 0.00 ^a^	0.17 ± 0.00 ^a^
Alanine	0.25 ± 0.02 ^a^	0.32 ± 0.01 ^a^	0.32 ± 0.01 ^a^
Cysteine	*n.d.*	*n.d.*	*n.d.*
Valine	0.21 ± 0.00 ^a^	0.26 ± 0.00 ^b^	0.25 ± 0.01 ^b^
Methionine	0.06 ± 0.01 ^a^	0.08 ± 0.00 ^b^	0.09 ± 0.01 ^b^
Isoleucine	0.12 ± 0.00 ^a^	0.16 ± 0.00 ^b^	0.17 ± 0.00 ^b^
Leucine	0.30 ± 0.00 ^a^	0.41 ± 0.01 ^b^	0.45 ± 0.00 ^c^
Tyrosine	0.04 ± 0.01 ^a^	0.09 ± 0.00 ^b^	0.07 ± 0.00 ^c^
Phenylalanine	0.16 ± 0.00 ^a^	0.21 ± 0.00 ^b^	0.21 ± 0.00 ^b^
Histidine	0.06 ± 0.00 ^a^	0.08 ± 0.00 ^b^	0.08 ± 0.01 ^b^
Lysine	0.12 ± 0.01 ^a^	0.13 ± 0.00 ^a^	0.15 ± 0.00 ^b^
Arginine	0.28 ± 0.00 ^a^	0.37 ± 0.01 ^b^	0.39 ± 0.00 ^c^
**∑**	3.3 ^a^	4.15 ^b^	4.25 ^c^

*n.d.—not detected.*

## Data Availability

The original contributions presented in the study are included in the article/Supplementary Material, further inquiries can be directed to the corresponding author.

## Supplementary Materials

The following supporting information can be downloaded at: https://www.mdpi.com/article/10.3390/foods14152668/s1, Table S1: The list of descriptors used for sensory evaluation.
