# Supplementary material for: Exploring Sorghum Flour as a Sustainable Ingredient in Gluten-Free Cookie Production
Source: Foods. 2025 Jul 29;14(15):2668. doi: 10.3390/foods14152668 (PMC12346720; doi:10.3390/foods14152668)
Supplement: Supplementary file 1 [file foods-14-02668-s001.zip › foods-3754808-supplementary.pdf]

SUPPLEMENTARY MATERIAL for

Exploring sorghum flour as a sustainable ingredient in gluten-free cookie production

By the authors: Simona Bukonja, Jelena Tomić, Mladenka Pestorić, Nikola Maravić, Saša Despotović, Zorica Tomičić, Biljana Kiprovski and Nebojša Đ. Pantelić

Table S1. The list of descriptors used for sensory evaluation

ATRIBUTE / DESCRIPTORS		
Appearance	Shape (visual)	Shape unevenness
	Surface (visual)	Surface unevenness
	Color	S2040-Y20R
		S1020-Y20R
		S0540-Y10R
		S5020-Y30R
		S4040-Y30R
		S4020-Y30R
		S4020-Y20R
		S0530-Y10R
		Color unevenness
	Texture—Manual	Hardness (palpatory)
		Crumbliness (visual)
		Pore unevenness (visual)
Odor	Nasal	Sweet
		Nutty
		Butter
		Milk
		Vegetable oil
		Coconut
		Sorghum
		Cocoa
Flavor (olfactory, gustatory, and trigeminal sensations in the mouth)	Taste (gustatory)	Sweet
		Bitter
	Flavor	Sweetness
		Sorghum
		Integral flour
		Starch
		Butter
		Vegetable oil
		Coconut

Astringency
Overall flavor intensity
Persistence
Aftertaste

Texture—Initial Bite

Fracturability
Hardness

Texture—Mouthfeel (oral)

Crunchiness
Granular
Toothpack
Hardness
Chewiness
Fracturability
Dryness
Oily
Chalky (aftertaste)
Oily Mouth Coating (aftertaste)
